# The Network Construction of a New Byproduct-Free XLPE-Based Insulation Using a Click Chemistry-Type Reaction and a Theoretical Study of the Reaction Mechanism

**DOI:** 10.3390/polym16243536

**Published:** 2024-12-19

**Authors:** Yang Du, Hui Zhang, Wei Han, Xia Du, Yan Shang, Hongda Yang, Xuan Wang, Qingguo Chen, Zesheng Li

**Affiliations:** 1 Key Laboratory of Engineering Dielectrics and Its Application of Ministry of Education & School of Material Science and Chemical Engineering, Harbin University of Science and Technology, Harbin 150080, China; duyang950711@163.com (Y.D.); charles_han@fjnu.edu.cn (W.H.); duxia62@126.com (X.D.); shangyan1972@126.com (Y.S.); qgchen@hrbust.edu.cn (Q.C.); 2 Electric Power Research Institute, State Grid Heilongjiang Electric Power Co., Ltd., Harbin 150030, China; yanghongda_phd16@hrbust.edu.cn; 3 Key Laboratory of Cluster Science of Ministry of Education & School of Chemistry, Beijing Institute of Technology, Beijing 100081, China; zeshengli@bit.edu.cn

**Keywords:** power cable, polyethylene, byproduct-free, cross-linking reaction mechanism, 1,5-disubtituted pentane, 2-ethylbutyric acid oxiranylmethyl ester

## Abstract

Cross-linked polyethylene (XLPE) is applied in most advanced high-voltage direct-current (HVDC) power cable insulations, which are produced via dicumyl peroxide (DCP) technology. The electrical conductivity of insulation material can be increased by cross-linking byproducts from the DCP process. Hence, currently much attention is being paid to a new process to produce cross-linking byproduct-free XLPE. The cross-linking in situ between ethylene–glycidyl methacrylate copolymer and 1,5-disubtituted pentane via reactive compounding is a substitute for DCP. The reaction potential energy information of the eighteen reaction channels was obtained at the B3LYP/6-311+G(*d*,*p*) level. Results demonstrated that epoxy groups and 1,5-disubtituted reactive groups can react in situ to realize the XLPE-based network structure via covalent linking, and epoxy ring openings yielded ester. 1,5-disubtituted pentane played a cross-linker role. The reactivity of the carboxyl group was stronger than that of the sulfydryl or hydroxyl group. The reaction channel RTS1 was more kinetically favorable due to the lower reaction Gibbs energy barrier height of 1.95 eV. The cross-linking network construction of the new XLPE insulation without byproducts opens up the possibility of DCP substitution, which is beneficial to furthering the design of thermoplastic insulation materials for power cables in the future.

## 1. Introduction

The transition from using fossil energy sources to renewables is urgently required for carbon emission reduction. Usually, wind and solar energy are most abundant in regions far away from end-users, which requires long-distance and efficient power transportation. Transmission lines need reverse energy flow direction because renewable energy sources are intermittent. High-voltage direct-current (HVDC) transmission lines meet these requirements, which will be a vital part of power grids in the future to integrate renewables seamlessly [1]. When transmission lines pass through densely populated areas or large bodies of water, it is necessary to submerge or bury power cables underground, and it is essential for transmission lines to be surrounded by insulation layers. A large amount of research is focused on upgrading the quality of insulated cables so as to increase transmission voltage outside the present ultimate limit (640 kV) [2]. Because at low temperatures polyethylene becomes too brittle and at 70 °C becomes too stiff, polyethylene by itself fails to be an appropriate insulation material. Therefore, developing the above all insulation for advanced high-voltage power cable involves obtaining cross-linked polyethylene (XLPE) with outstanding electrical and mechanical behaviors.

Recently, several strategies, such as grafting a voltage stabilizer or antioxidant onto polyethylene, introducing deep traps, and modifying montmorillonite, have been proposed as means to improve electrical tree resistance [3], thermal aging properties [4], and space charge behavior [5]. In order to improve material properties [6] and enhance material functionality [7], nanotechnology and organic–inorganic hybrids are often used as effective methods.

However, XLPE production often uses dicumyl peroxide (DCP) [8] as a cross-linker, which does lead to the emission of some volatile or hazardous byproducts [9] (methane, acetophenone, cumyl alcohol, *a*-methyl styrene, etc.,) and usually needs a time- and energy-consuming degassing procedure to remove these unwanted byproducts [10]. Even trace amounts of residual impurities can raise the electrical conductivity of polyethylene [11]. The overall quality of XLPE insulation improves with any reduction in the amount of additives used. Obviously, it would be desirable to find preparation strategies for polyethylene copolymer blends that can replace DCP cross-linking and entirely prevent the emission of harmful byproducts, and thereby improve the total quality of the insulation material.

Müller and colleagues prepared XLPE insulation via blending two polyethylene copolymers, p(E-stat-GMA) (ethylene–glycidyl methacrylate copolymer) and p(E-stat-AA) (ethylene–acrylic acid copolymer) [12]. The epoxy and carboxyl groups, respectively, from these two polyethylene copolymers react to form covalent bonds at 160 °C. The resulting cross-linking product meets the demands of polyethylene resin, and at the same time, its electrical conductivity is equivalent to the measured value of commercial XLPE. Müller’s research group explored the preparation process of a polyethylene–polypropylene-type (PE-PP-type) copolymer in situ blended at 170 °C [13], and confirmed their recyclability via a reprocessing process at the same temperature. Covalent bonds form between two polyethylene copolymers: one is p(E-stat-GMA) and the other is maleic anhydride-grafted polypropylene (PP-graft-MA). They also proved that polyethylene copolymers containing epoxy resin and glycidyl methacrylate can be efficiently cross-linked with three bifunctional curing agents (amines, carboxylic acids, and hydrazides), without byproduct formation, at 160 to 200 °C [14]. This promising approach inspired us to explore new cross-linking network construction of thermoplastic insulation materials for power cables.

In this study, the design of a new reaction system to produce XLPE in situ without additives or the release of any byproducts was established. The cross-linking reaction in situ between epoxy groups with 1,5-disubtituted reactive groups replaced the DCP cross-linking process, and electronic effects were estimated. Molecular formulas, molecular names, and corresponding abbreviations of 1,5-disubtituted pentane molecules are listed in Table 1. 1,5-disubtituted pentane can be used as a cross-linker.

As far as we know, theoretical research on the reaction mechanisms of the synergistic cross-linking reaction between epoxy groups and 1,5-disubtituted reactive groups at molecular and atomic levels has been rarely reported. These reaction processes are described in detail. The novel proposed reaction mechanism would be beneficial for opening up the possibility of cross-linking network construction and expanding the applicative range of polyethylene-based insulation materials for advanced power cables in the future.

## 2. Computational Methods

The density functional theory (DFT) [14] was used to study the reaction mechanism of the synergistic cross-linking reaction. At the B3LYP/6-311+G(*d*,*p*) level [15], geometry structures were optimized, and along the minimum Gibbs free energy path (MEP) [16], energies, gradients and force constants of reactants, transition states, and products were calculated. According to the results of these stationary electronic structure calculations, potential energy surface information was obtained. This research method has been proven reliable and suitable for current research [17]. Therefore, our objective was to study the reaction mechanism of the synergistic cross-linking reaction and estimate the electronic effects of substituted groups. When a gradient step-size was 0.05 (amu)^1/2^ bohr, the MEP was achieved by intrinsic reaction coordinate (IRC) theory [18]. The transition state and reaction channel were abbreviated to the corresponding TS and R, separately. These calculations of electronic structures were completed by the GAUSSIAN09 program package [19].

## 3. Results and Discussion

### 3.1. Stationary Point Geometries

**Figure 1:**
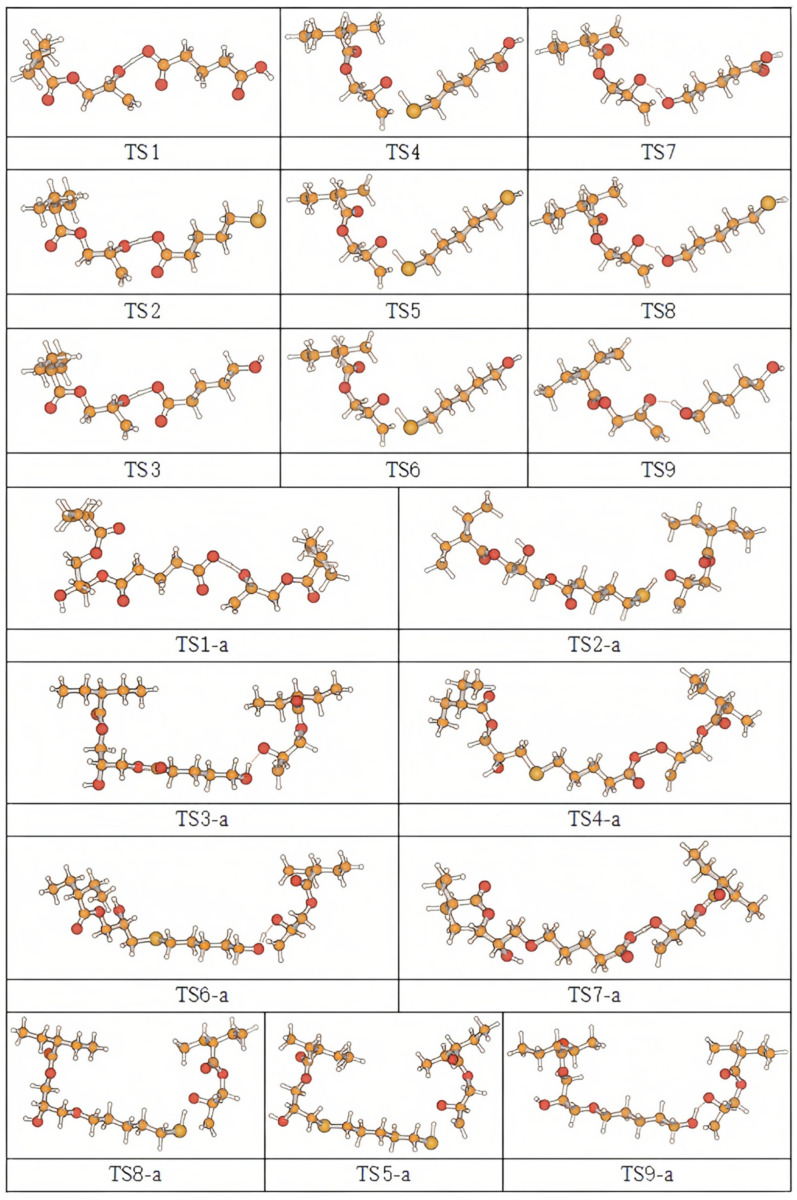
Optimized geometric structures of the eighteen transition states.

In this work, theoretical study of the reaction behavior of a synergistic cross-linking reaction was completed at the B3LYP/6-311+G(*d*,*p*) level. In Table 2, reaction equations for the eighteen studied reaction channels are plotted; 2-ethylbutyric acid oxiranylmethyl ester was selected as a model molecule of an ethylene–glycidyl methacrylate copolymer. The labels of transition states and reaction channels correspond one-to-one. In Figure 1, geometry structures of the eighteen transition states after optimization are provided. In Electronic Supplementary Materials (ESM), the corresponding standard orientations are offered. Table 2 also lists the bond lengths (b/f) for transition state cleavage and bond formation after optimization, the corresponding bond lengths in the equilibrium geometries of reactants and products, and the imaginary frequency values (freq.) for transition states. For the transition state TS1, the length of the breaking bond (b) O-H on the carboxyl group was 1.233 Å, and the lengths of forming bonds (f) O-H and O-C were 1.169 and 2.299 Å. As seen in Table 2, for synergistic reaction channels TS1, TS2, and TS3, the structures of transition states shared common characteristics. In the equilibrium molecules, compared with the elongation of the forming bonds, the elongation of the breaking bonds was relatively smaller, separately. That is to say, the hydrogen abstraction reactions were all reactant-like. These reaction channels proceeded via “early” transition states and applied to an exothermic reaction, which is in accordance with Hammond’s postulate [20].

### 3.2. The Minimum Gibbs Free Energy Path

**Figure 2:**
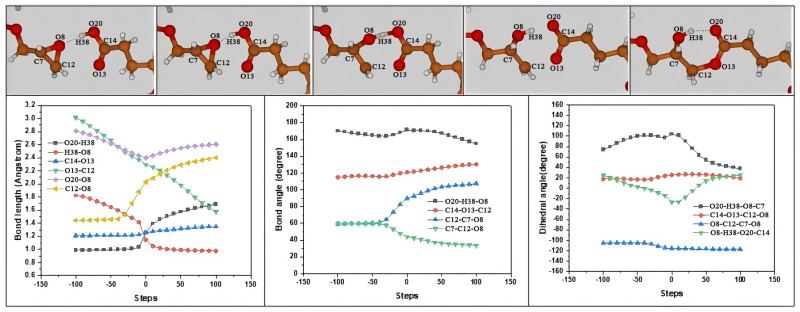
Changes in bond lengths, bond angles, and dihedral angles of reaction channel R1 along minimum Gibbs free energy path (MEP).

In Figure 2, three key parameters along the MEP, including bond lengths, bond angles, and dihedral angles of reaction channel R1 at the B3LYP/6-311+G(*d*,*p*) level, are depicted. The synergistic reaction channel (R1) involves the H-transfer of a carboxyl group to O on epoxy and the O-attack of a carboxylic group to a C atom of the -CH- site on epoxy via transition state TS1, at time step 0. From reactant (step −100) to product (step 100), five key steps are included here.

First, at IRC step −100, the H of the carboxyl group transferred to an O atom on epoxy; the bond length of O20-H38 on the carboxyl group was 0.987 Å; it was 3.012 Å between O13 on the carbonyl group and C12 in the -CH_2_- site on epoxy; the distance between H38 and O8 on epoxy was 1.828 Å; and the bond length of C12-O8 on epoxy was 1.444 Å. Second, at IRC step −30, the H38 gradually transferred to the O8 atom; the distance between H38 and O8 decreased to 1.539 Å; and at the same time the epoxy ring opened, the distance between O13 and C12 was 2.483 Å and the distance between C12 and O8 was 1.528 Å. Third, at IRC step 0, the transition state TS1 presented stretch oscillating of H38 between O8 and O20; the O13 atom continued to approach C12; and O8, H38, and O20 nearly became a straight line, ∠O20-H38-O8, which peaked at 171.42°. Meanwhile H38, O20, C14, O13, O8, and C12 participated in the reaction and formed a hexatomic ring structure, which was almost co-planed. Fourth, at IRC step 25, the H38 transferred to the O8 atom, the bond of O20 and H38 broke, the bond length of H38-O8 continued to decrease to 1.011 Å, and it was 2.178 Å between O13 and C12. Last, at IRC step 100, O13 attacked the C12 atom to form an O-C bond. Now, with a bond length of 1.576 Å, the distance between the ring-opened C12 and O8 was 2.400 Å, while the bond length of the carbonyl group O13-C14 almost remained unchanged throughout the entire reaction process.

### 3.3. Energetics

In Table 1, at the B3LYP/6-311+G(*d*,*p*) level, the reaction Gibbs free potential barrier heights (Δ*G*^≠^) and the reaction Gibbs free energies (Δ*G*) at 298 K of the eighteen reaction channels calculated are presented. For the reaction of epoxy alcohol with carboxylic acid, the reaction of ring-opening and cyclization of epoxy alcohol can be catalyzed by the carboxylic acid [21]. For the reaction of epoxy groups in epoxy resin containing amino groups, it is generally agreed to generate new C-N and O-H groups throughout the curing reaction process [22,23]. The synergistic cross-linking reaction refers to this process, in which the hydrogen in active functional groups (a hydroxyl or sulfydryl group) transfers to the oxygen on epoxy, and meanwhile the oxygen of the carbonyl group (or the oxygen of the hydroxyl group or the sulfur of the sulfydryl group) attacks the carbon of -CH_2_- on epoxy.

As seen in Table 1, the synergistic cross-linking reaction between epoxy groups and 1,5-disubtituted reactive groups was a nucleophilic addition reaction. Studied 1,5-disubtituted (bifunctional groups) pentanes (PDA, HPA, MPA, PDOL, MPOL, and PDM) can be used as the attacking reagent. One side functional group reacts with the epoxy compound, and then the other side functional group also undergoes nucleophilic addition, with another molecule epoxy compound being considered.

First, for reactions 1, 4 and 7, one side functional group (-COOH, -SH, or OH) reacted with epoxy compound, and the other side functional group was a carboxyl group. The reaction Gibbs free energy barriers of reaction channel R1 (the one side functional group was -COOH) was the lowest (Δ*G*^≠^_R1_ = 1.95 eV), followed by reaction channels R4 (the one side functional group was -OH) (Δ*G*^≠^_R4_ = 2.45 eV) and R7 (the one side functional group was -SH) (Δ*G*^≠^_R7_ = 2.54 eV). According to the report, it was easier for the carboxyl group to provide hydrogen for the protonation of the epoxy group [21]. Because the stronger the acidity, the higher the reactivity, the lower the value of Δ*G*^≠^. The acidity of the -COOH group was the strongest among the three kinds of substituted groups (-COOH, -SH, and OH); thus, Δ*G*^≠^ (R1) was the lowest, and the reaction was the easiest. Similar reactivity rules were also found when the other side functional groups were hydroxyl and sulfydryl groups.

**Figure 3:**
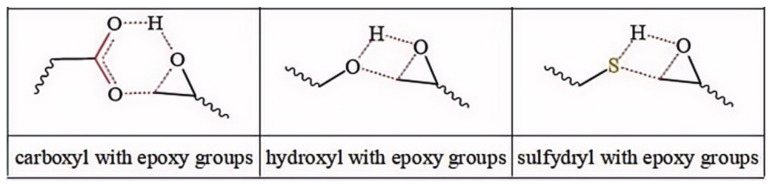
Schematic diagrams of synergistic cross-linking reaction of functional groups with epoxy groups.

Meanwhile, for reactions 4 and 7, the active functional groups (-SH and -OH) attacked the O atom on the epoxy group (see Figure 3), and the O-H bond of the -OH or the S-H bond of -SH broke. The active H of active functional groups transferred to O on the epoxy group. The oxygen atom of -OH or the sulfur atom of -SH attacked the carbon atom in -CH_2_- sites on the epoxy group. Tetra-atomic ring structures were exhibited on transition states TS4 and TS7, and these structures were unstable; because of their large amounts of tension, they possessed higher energy. In reaction 1, the active H and acyl O in carboxyl groups attacked the oxygen and carbon atoms in the epoxy group; separately, a hex-atomic ring structure was exhibited on the transition state TS1, the structure was almost co-plane and nearly non tension, and the structure of the transition state was more stable. Thus, the reaction Gibbs free potential barriers of TS1 were lower than those of TS4 and TS7.

Second, for reactions 7-a, 8-a, and 9-a, after one side functional group (OH) reacted with the epoxy group compound, the other side functional groups (-COOH, -SH, or OH) underwent nucleophilic addition with another molecule epoxy compound. The reaction Gibbs free energy barriers of reaction channel R7-a was the lowest (Δ*G*^≠^_R7-a_ = 2.25 eV), followed by reaction channels R9-a (Δ*G*^≠^_R9-a_ = 2.49 eV) and R8-a (Δ*G*^≠^_R8-a_ = 2.66 eV). Because the volume of the hydroxyl group was relatively small, the reactivity mainly depended on the acidity of functional groups. The cross-linking reaction was more likely to occur due to the lowest Δ*G*^≠^ (R7-a).

Third, for reactions 4-a, 5-a, and 6-a, after one side functional group (SH) reacted with the epoxy group compound, the other side functional groups (-COOH, -SH, or OH) reacted with another molecule epoxy compound. The reaction Gibbs free energy barrier was affected by two main factors. On the one hand, the larger the volume of the attacking reagent, the greater the steric hindrance, and the reaction proceeded with difficulty. On the other hand, it was determined by the acidity of the reagent, which was strong, and the reaction was prone to occur. The hydroxyl group possessed the smallest size, the carboxyl group had the strongest acidity, and comprehensively considering these two factors, as a result, the reaction Gibbs free energy barriers followed the trend Δ*G*^≠^_R6-a_ (2.55 eV) < Δ*G*^≠^_R4-a_ (2.61 eV) < Δ*G*^≠^_R5-a_ (2.81 eV). Eslami and Müller-Plathe [24] reported that free-energy barriers for the nucleation of different crystals are dramatically different, and a high energy barrier may be due to the rearrangement of particles during the crystalline process. In this study, the network construction of insulation materials was carried out through melt blending. However, this method involves the process of cooling and extrusion molding, which can form an amorphous structure. This amorphous structure can lead to uneven crystallization, with no lattice forming. In the next step, we will try to further study two molecules of 1,5-disubstituted pentane participated reactions.

Müller and colleagues established a new preparation process using the blending reaction of two polyethylene copolymers at 160 °C [12]. They explored a recycle approach for the in situ formation of a polyethylene–polypropylene-type copolymer via ternary blending at 170 °C [13]. They also demonstrated the efficient cross-linking of an ethylene–glycidyl methacrylate copolymer with bifunctional curing agents (amine, carboxylic acids, or hydrazides) at 160 to 200 °C [14]. Our theoretical calculation results demonstrate that the nucleophilic addition reaction channel RTS1 epoxy-bearing polyethylene copolymer and -COOH group of 1,5-disubtituted pentane (PDA) is more favorable with lower reaction Gibbs free energy barriers. Synergistic cross-linking forms covalent bonds with a click chemistry-type reaction.

## 4. Conclusions

In this study, a new cross-linking network of XLPE-based insulation was constructed with a click chemistry-type reaction based on theoretical calculation results of density functional theory, and a theoretical study of the cross-linking reaction mechanism between epoxy-bearing polyethylene copolymer and 1,5-disubtituted pentane was proposed for the first time. The reactivity of the carboxyl group was the highest, and stronger than those of hydroxyl and sulfydryl groups. The reaction channel R1 of epoxy-bearing polyethylene copolymer and carboxyl group substitution diacid PDA was conducive to the progress of the crosslinking reaction with the reaction Gibbs energy barrier as low as about 1.95 eV at the B3LYP/6-311+G(*d*,*p*) level. Cross-linking reactions processed in situ underwent epoxy ring-opening and nucleophilic addition reaction. Evidently, the designed cross-linking enabled the realization of a new type of network XLPE-based insulation material, with an atomic economic benefit of 100%. The use of reactive compounding with click chemistry is a promising approach, which sets up a byproduct-free alternative to conventional peroxides cross-linking. Further research is underway on electron injection and storage behavior in novel network XLPE-based insulating materials.

## Figures and Tables

**Table 1:** Molecular names, molecular formulas, and corresponding abbreviations (ab.) of 1,5-disubtituted pentane molecules.

Molecular Formula	Molecular Name	ab.
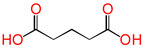	glutaric acid	PDA
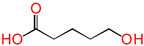	5-hydroxypentanoic acid	HPA
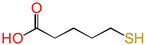	5-mercaptopentanoic acid	MPA
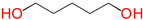	1,5-pentanediol	PDOL
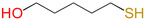	5-mercaptopentanol	MPOL
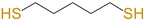	1,5-pentanedithiol	PDM

**Table 2:** Optimized bond lengths of breaking/forming bonds (b/f) for transition state, reactants (r) and products (p) (in Å), together with calculated breaking/forming bond frequencies (in cm^−1^), calculated reaction Gibbs free energies (Δ*G*), and Gibbs potential barrier heights (Δ*G*^≠^) (in eV).

Number	Reaction Equation	r	b/f	p	freq.	Δ*G*^≠^	Δ*G*
1	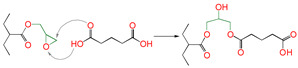	0.969	1.233/OH1.169 1.233/OC2.299	OH0.963 OC1.445	874 *i*	1.95	−0.36
1-a	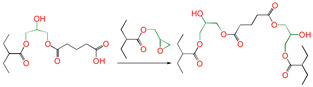	0.969	1.184/OH1.214 1.184/OC2.278	OH0.963 OC1.440	880 *i*	2.67	0.27
2	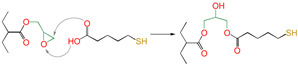	0.969	1.244/OH1.160 1.244/OC2.306	OH0.964 OC1.441	874 *i*	2.05	−0.42
2-a	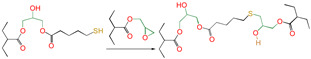	1.348	1.454/OH1.499 1.454/SC2.766	OH0.970 SC1.832	815 *i*	2.59	−0.64
3	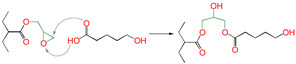	0.969	1.219/OH1.181 1.219/OC2.296	OH0.963 OC1.445	898 *i*	2.13	−0.41
3-a	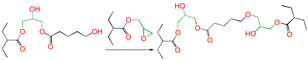	0.962	1.017/OH1.553 1.017/OC2.184	OH0.964 OC1.414	407 *i*	2.53	−0.37
4	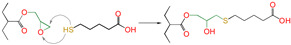	1.349	1.454/OH1.503 1.454/SC2.763	OH0.970 SC1.832	797 *i*	2.54	−0.64
4-a	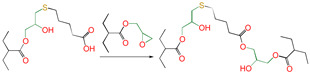	0.969	1.224/OH1.176 1.224/OC2.299	OH0.963 OC1.441	892 *i*	2.61	0.12
5	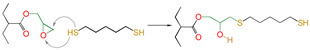	1.349	1.442/OH1.533 1.442/SC2.748	OH0.970 SC1.831	728 *i*	2.63	−0.62
5-a	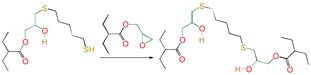	1.349	1.434/OH1.557 1.434/SC2.737	OH0.970 SC1.832	674 *i*	2.81	−0.51
6	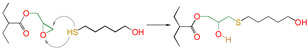	1.349	1.435/OH1.554 1.435/SC2.742	OH0.967 SC1.842	670 *i*	2.62	−0.66
6-a	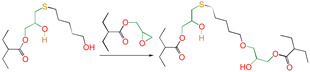	0.962	1.017/OH1.553 1.017/OC2.183	OH0.964 OC1.414	406 *i*	2.55	−0.37
7	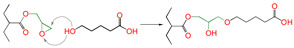	0.962	1.018/OH1.547 1.018/OC2.183	OH0.967 OC1.412	406 *i*	2.45	−0.40
7-a	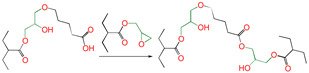	0.969	1.233/OH1.169 1.233/OC2.302	OH0.963 OC1.440	877 *i*	2.25	−0.12
8	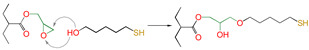	0.962	1.017/OH1.550 1.017/OC2.184	OH0.963 OC1.413	405 *i*	2.43	−0.47
8-a	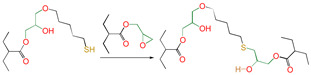	1.349	1.429/OH1.572 1.429/SC2.729	OH0.964 SC1.832	645 *i*	2.66	−0.63
9	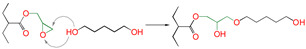	0.962	1.016/OH1.555 1.016/OC2.184	OH0.967 OC1.442	403 *i*	2.42	−0.39
9-a	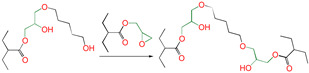	0.962	1.017/OH1.551 1.017/OC2.181	OH0.970 OC1.413	410 *i*	2.49	−0.44

## Data Availability

Original contributions presented in this study are included in this article and Supplementary Materials. Further inquiries can be directed to the corresponding authors.

## Supplementary Materials

The following supporting information can be downloaded at: https://www.mdpi.com/article/10.3390/polym16243536/s1, Text S1: The optimized standard orientations of transition states of the eighteen reaction channels at the B3LYP/6-311+G(*d*,*p*) level.
