# Supplementary material for: The Network Construction of a New Byproduct-Free XLPE-Based Insulation Using a Click Chemistry-Type Reaction and a Theoretical Study of the Reaction Mechanism
Source: Polymers (Basel). 2024 Dec 19;16(24):3536. doi: 10.3390/polym16243536 (PMC11678825; doi:10.3390/polym16243536)
Supplement: Supplementary file 1 [file polymers-16-03536-s001.zip › polymers-3335809-supplementary.pdf]

Supplementary Materials

The Network Construction of a New Byproduct-Free XLPE-Based Insulation Using a Click Chemistry-Type Reaction and a Theoretical Study of the Reaction Mechanism

Yang Du [1], Hui Zhang [1,*], Wei Han [1], Xia Du [1], Yan Shang [1], Hongda Yang [2], Xuan Wang [1,*], Qingguo Chen [1] and Zesheng Li [3]

[1] Key Laboratory of Engineering Dielectrics and Its Application of Ministry of Education & School of Material Science and Chemical Engineering, Harbin University of Science and Technology, Harbin 150080, China; duyang950711@163.com (Y.D.); charles_han@fjnu.edu.cn (W.H.); duxia62@126.com (X.D.); shangyan1972@126.com (Y.S.); qgchen@hrbust.edu.cn (Q.C.)
[2] Electric Power Research Institute, State Grid Heilongjiang Electric Power Co., Ltd., Harbin 150030, China; yanghongda_phd16@hrbust.edu.cn
[3] Key Laboratory of Cluster Science of Ministry of Education & School of Chemistry, Beijing Institute of Technology, Beijing 100081, China; zeshengli@bit.edu.cn
* Correspondence: hust_zhanghui11@hotmail.com (H.Z.); wangxuan@hrbust.edu.cn (X.W.)

The optimized standard orientations of transition states of the eighteen reaction channels at the B3LYP/6-311+G(*d,p*) level.

TS1

6	0	-5.444013	2.383946	-1.015092
6	0	-5.229785	1.770232	0.371667
6	0	-5.492485	0.249136	0.435890
6	0	-4.578076	-0.501993	-0.516532
8	0	-3.275564	-0.403594	-0.143004
6	0	-2.320588	-1.042523	-1.002038
6	0	-0.949927	-0.766767	-0.414055

8	0	-0.508662	0.557061	-0.634606
6	0	-5.379060	-0.287565	1.878383
6	0	-5.718441	-1.773873	2.023000
8	0	-4.926437	-1.115969	-1.495370
6	0	0.199599	-1.261938	-1.172989
8	0	2.243313	-0.269324	-0.824069
6	0	2.383469	0.623661	0.042620
6	0	3.782244	1.090538	0.418871
6	0	4.899148	0.095689	0.100096
6	0	6.273968	0.658780	0.458675
6	0	7.404998	-0.313709	0.220414
8	0	7.299561	-1.467557	-0.110730
8	0	1.413309	1.206143	0.638980
8	0	8.612283	0.271068	0.437547
1	0	-5.291157	3.465828	-0.985533
1	0	-6.458695	2.198361	-1.379654
1	0	-4.748022	1.975445	-1.753062
1	0	-4.210934	1.970213	0.716694
1	0	-5.904792	2.247904	1.089940
1	0	-6.059830	0.302590	2.501212
1	0	-4.368791	-0.094609	2.251416
1	0	-5.026701	-2.405583	1.457698

1	0	-5.665378	-2.084284	3.069799
1	0	-6.728719	-1.987561	1.661537
1	0	-6.502869	0.051501	0.063827
1	0	-2.392195	-0.630098	-2.010764
1	0	-2.523261	-2.117033	-1.045228
1	0	-0.927441	-1.050863	0.644087
1	0	0.223363	-1.077924	-2.238341
1	0	0.952117	-1.913675	-0.760854
1	0	0.375051	0.885586	0.057146
1	0	3.938579	2.033965	-0.119819
1	0	3.761432	1.352026	1.480863
1	0	4.734739	-0.837174	0.645527
1	0	4.868567	-0.167346	-0.958847
1	0	6.495292	1.571160	-0.105842
1	0	6.321933	0.951769	1.514548
1	0	9.288990	-0.404515	0.280207

TS2

6	0	-4.918307	-1.144746	2.691730
6	0	-4.616368	-1.879823	1.382719
6	0	-5.227150	-1.218731	0.127651
6	0	-4.722978	0.203620	-0.044919

8	0	-3.381819	0.238137	-0.259801
6	0	-2.796037	1.540816	-0.394310
6	0	-1.301446	1.335788	-0.546104
8	0	-0.665501	0.993030	0.669031
6	0	-4.976430	-2.067116	-1.137191
6	0	-5.627396	-1.511953	-2.407449
8	0	-5.397543	1.203309	-0.001151
6	0	-0.476427	2.542847	-0.610553
8	0	1.747768	2.404094	-0.028225
6	0	2.263923	1.301587	0.271296
6	0	3.774553	1.225944	0.431860
6	0	4.394965	-0.071479	-0.100799
6	0	5.909127	-0.130612	0.138303
6	0	6.526576	-1.420390	-0.400824
1	0	6.351581	-1.506243	-1.474988
8	0	1.596406	0.229551	0.473843
16	0	8.349398	-1.412483	-0.093558
1	0	-4.501964	-1.688197	3.543729
1	0	-5.995859	-1.043509	2.852395
1	0	-4.486066	-0.140201	2.702388
1	0	-3.534502	-1.969052	1.246167
1	0	-5.011679	-2.899883	1.438338

1	0	-5.365265	-3.071563	-0.938802
1	0	-3.897435	-2.174596	-1.284739
1	0	-5.229719	-0.527369	-2.670800
1	0	-5.448751	-2.176364	-3.256943
1	0	-6.709666	-1.407151	-2.286845
1	0	-6.306964	-1.113681	0.273778
1	0	-3.010006	2.139401	0.493613
1	0	-3.215759	2.046218	-1.269551
1	0	-1.094510	0.620904	-1.350200
1	0	-0.668803	3.331998	0.103186
1	0	0.215371	2.749186	-1.410142
1	0	0.401230	0.541486	0.541688
1	0	4.212440	2.105177	-0.045067
1	0	3.971916	1.315390	1.507591
1	0	3.903314	-0.923327	0.376119
1	0	4.191147	-0.155507	-1.174769
1	0	6.393611	0.729804	-0.337207
1	0	6.114085	-0.052083	1.212008
1	0	8.608569	-2.622777	-0.628880
1	0	6.089210	-2.287821	0.096820

TS3

6	0	-4.012907	2.546203	-1.845548
6	0	-4.032212	2.363572	-0.325338
6	0	-4.827702	1.130802	0.156665
6	0	-4.248084	-0.152855	-0.413124
8	0	-2.979978	-0.365757	0.023991
6	0	-2.319948	-1.533265	-0.486318
6	0	-0.894326	-1.489129	0.030155
8	0	-0.093999	-0.538784	-0.640696
6	0	-4.910552	1.079499	1.697231
6	0	-5.755656	-0.074057	2.245233
8	0	-4.811305	-0.912330	-1.163131
6	0	0.002399	-2.549064	-0.433098
8	0	2.266941	-2.169608	-0.410295
6	0	2.737017	-1.109633	0.064958
6	0	4.229691	-1.032498	0.342515
6	0	4.865194	0.315520	-0.018686
6	0	6.354480	0.368112	0.337740
6	0	6.996793	1.696233	-0.032666
1	0	6.487146	2.517949	0.489305
8	0	2.030310	-0.083988	0.362409
8	0	8.376126	1.642843	0.342510
1	0	-3.476090	3.458670	-2.117294

1	0	-5.026665	2.619840	-2.250598
1	0	-3.515225	1.712389	-2.348728
1	0	-3.009050	2.297683	0.056115
1	0	-4.485320	3.245595	0.140316
1	0	-5.334974	2.031801	2.032795
1	0	-3.896661	1.029465	2.105873
1	0	-5.336418	-1.048501	1.977581
1	0	-5.811875	-0.029301	3.336041
1	0	-6.777460	-0.038592	1.856006
1	0	-5.842802	1.182370	-0.249755
1	0	-2.330100	-1.520778	-1.578043
1	0	-2.838935	-2.432028	-0.138590
1	0	-0.889042	-1.358055	1.118322
1	0	0.003392	-2.791546	-1.486856
1	0	0.553994	-3.195271	0.229439
1	0	0.910052	-0.264265	-0.083093
1	0	4.351920	-1.220710	1.417112
1	0	4.715218	-1.858384	-0.180846
1	0	4.735438	0.496105	-1.092407
1	0	4.321455	1.111774	0.496491
1	0	6.490248	0.204614	1.412699
1	0	6.898146	-0.433799	-0.173866

1	0	8.795302	2.476026	0.108313
1	0	6.898426	1.871308	-1.112931

TS4

6	0	1.941692	-2.213473	1.298260
6	0	3.402540	-1.814313	1.528672
6	0	4.232960	-1.676103	0.234440
6	0	5.696202	-1.288177	0.536797
6	0	6.606491	-1.254635	-0.694424
6	0	3.589594	-0.702021	-0.738709
8	0	3.207763	-0.975062	-1.849021
8	0	3.505458	0.546060	-0.200698
6	0	2.795724	1.554563	-0.933069
6	0	1.675892	2.073314	-0.027282
6	0	0.723369	2.980582	-0.677794
8	0	0.703267	1.109873	0.241082
16	0	-1.478042	2.816785	0.983911
6	0	-2.640685	1.904420	-0.117822
6	0	-3.435911	0.830224	0.627041
6	0	-4.424236	0.102806	-0.294772
6	0	-5.203625	-0.985121	0.443247
6	0	-6.207505	-1.703743	-0.426997

8	0	-6.833227	-2.696402	0.257081
1	0	-7.461724	-3.111565	-0.352650
1	0	-3.309077	2.650423	-0.552509
1	0	5.709023	-0.315709	1.038777
1	0	6.085592	-2.015967	1.257098
1	0	7.633782	-1.010370	-0.410783
1	0	6.621333	-2.222829	-1.203627
1	0	6.278425	-0.506682	-1.422019
1	0	4.226165	-2.636021	-0.291903
1	0	3.445030	-0.869786	2.079546
1	0	3.898737	-2.565881	2.152927
1	0	1.869555	-3.139179	0.718512
1	0	1.435023	-2.378257	2.253327
1	0	1.392934	-1.431423	0.766171
1	0	3.505618	2.349593	-1.179054
1	0	2.384850	1.121993	-1.845362
1	0	2.119810	2.506330	0.881747
1	0	0.516243	3.989199	-0.354061
1	0	0.273628	2.645888	-1.603657
1	0	-0.432644	1.808029	0.935706
1	0	-2.054835	1.456356	-0.922816
1	0	-3.974782	1.291395	1.461882

1	0	-2.737126	0.110037	1.065980
1	0	-5.128593	0.818665	-0.729864
1	0	-3.888566	-0.345888	-1.137119
1	0	-4.531560	-1.739557	0.866612
1	0	-5.751550	-0.570465	1.297375
8	0	-6.457215	-1.460321	-1.580733

TS5

6	0	2.139119	-2.070233	1.651712
6	0	3.639556	-1.762513	1.658899
6	0	4.300146	-1.779349	0.264858
6	0	3.634091	-0.798111	-0.686110
8	0	3.714946	0.475626	-0.209593
6	0	3.025654	1.513307	-0.919486
6	0	1.939994	2.069279	0.008681
8	0	0.939695	1.140806	0.290982
6	0	1.016912	3.017079	-0.626114
6	0	5.820381	-1.523287	0.357796
6	0	6.565030	-1.635710	-0.975638
8	0	3.118452	-1.085134	-1.736888
16	0	-1.222896	2.913370	0.963242
6	0	-2.338832	2.048674	-0.222711

6	0	-3.313584	1.090256	0.465193
1	0	-2.880566	2.826744	-0.764767
1	0	5.986683	-0.534341	0.796471
1	0	6.231207	-2.251115	1.066013
1	0	7.638261	-1.482010	-0.833948
1	0	6.425284	-2.622396	-1.427317
1	0	6.218050	-0.892327	-1.699047
1	0	4.137656	-2.760870	-0.192160
1	0	3.815127	-0.787037	2.122698
1	0	4.164699	-2.501529	2.274673
1	0	1.934178	-3.035847	1.178569
1	0	1.752110	-2.111962	2.673691
1	0	1.575538	-1.300430	1.116991
1	0	3.762764	2.281361	-1.169718
1	0	2.582129	1.102391	-1.826644
1	0	2.417508	2.482118	0.910436
1	0	0.857673	4.032669	-0.296400
1	0	0.550203	2.710122	-1.553100
1	0	-0.216260	1.881124	0.972482
1	0	-1.705991	1.509372	-0.930386
6	0	-4.230207	0.382313	-0.542481
1	0	-3.918665	1.643860	1.190882

1	0	-2.746544	0.345589	1.033208
6	0	-5.228958	-0.569654	0.129188
1	0	-4.779477	1.132569	-1.125260
1	0	-3.617045	-0.178229	-1.258689
6	0	-6.126866	-1.278123	-0.884226
1	0	-4.684546	-1.317099	0.716520
1	0	-5.850609	-0.007492	0.835183
16	0	-7.323325	-2.371827	0.004354
1	0	-6.687488	-0.548541	-1.472034
1	0	-5.527919	-1.889696	-1.561571
1	0	-7.933775	-2.865637	-1.091665

TS6

6	0	2.002419	2.121441	-1.598725
6	0	3.458691	1.648053	-1.632841
6	0	4.140006	1.567652	-0.250671
6	0	3.378407	0.658703	0.700385
8	0	3.306388	-0.610053	0.210806
6	0	2.515223	-1.572648	0.920988
6	0	1.383648	-2.020366	-0.010811
8	0	0.478194	-1.002172	-0.296749
6	0	0.376686	-2.880009	0.622544

6	0	5.616720	1.134336	-0.377601
6	0	6.392340	1.129596	0.943050
8	0	2.912494	0.992646	1.760722
16	0	-1.831025	-2.589480	-0.978040
6	0	-2.881676	-1.641795	0.204098
6	0	-3.817648	-0.648699	-0.488372
1	0	-3.454159	-2.378750	0.771641
1	0	5.655472	0.142461	-0.838689
1	0	6.099440	1.823167	-1.079521
1	0	7.438201	0.857275	0.777104
1	0	6.374569	2.115180	1.417671
1	0	5.974557	0.414363	1.657409
1	0	4.103259	2.554740	0.221122
1	0	3.517010	0.666564	-2.113265
1	0	4.054725	2.333032	-2.246678
1	0	1.912336	3.092490	-1.101627
1	0	1.611752	2.229967	-2.614347
1	0	1.361594	1.407660	-1.073568
1	0	3.171613	-2.409143	1.177293
1	0	2.110544	-1.117317	1.825033
1	0	1.823463	-2.477829	-0.910662
1	0	0.132973	-3.880750	0.299212

1	0	-0.066714	-2.527026	1.544649
1	0	-0.753404	-1.642377	-0.994414
1	0	-2.209309	-1.122361	0.889573
6	0	-4.676150	0.128422	0.519591
1	0	-4.464963	-1.184702	-1.190393
1	0	-3.224144	0.053001	-1.083246
6	0	-5.638859	1.113821	-0.152654
1	0	-5.247184	-0.581244	1.132112
1	0	-4.017492	0.670576	1.209587
6	0	-6.471329	1.899545	0.849029
1	0	-5.079243	1.823018	-0.771795
1	0	-6.318291	0.577068	-0.823563
8	0	-7.341441	2.768037	0.119220
1	0	-7.053872	1.209829	1.476235
1	0	-5.812821	2.480263	1.509952
1	0	-7.859465	3.286540	0.741754

TS7

6	0	-2.421351	-2.526554	-0.714321
6	0	-3.806812	-1.994926	-1.093961
6	0	-4.597874	-1.381911	0.081405
6	0	-3.807520	-0.285243	0.776414

8	0	-3.519958	0.730902	-0.081877
6	0	-2.654692	1.781523	0.372904
6	0	-1.432830	1.799371	-0.551835
8	0	-0.657962	0.648463	-0.463768
6	0	-0.352219	2.692870	-0.103536
6	0	-5.983520	-0.875075	-0.373131
6	0	-6.870511	-0.354994	0.761827
8	0	-3.483187	-0.289685	1.937659
8	0	1.443271	1.819552	-0.986147
6	0	2.535735	1.557763	-0.102291
6	0	3.510799	0.554526	-0.717307
1	0	3.038313	2.512487	0.078098
1	0	-5.844875	-0.094034	-1.127290
1	0	-6.487463	-1.707845	-0.875906
1	0	-7.848726	-0.049662	0.380495
1	0	-7.033346	-1.123672	1.523220
1	0	-6.425771	0.510987	1.260498
1	0	-4.739587	-2.148732	0.849875
1	0	-3.709358	-1.244967	-1.884997
1	0	-4.416744	-2.807675	-1.504494
1	0	-2.484970	-3.256122	0.099381
1	0	-1.955170	-3.022658	-1.570312

1	0	-1.755516	-1.717688	-0.400698
1	0	-3.215205	2.719275	0.312205
1	0	-2.356766	1.585861	1.403318
1	0	-1.761848	2.024701	-1.578707
1	0	0.035095	3.522307	-0.678489
1	0	-0.038377	2.604500	0.929237
1	0	0.754671	1.072122	-0.931757
1	0	2.159156	1.179814	0.857037
6	0	4.719521	0.293411	0.188881
1	0	3.839133	0.938443	-1.689215
1	0	2.975607	-0.382170	-0.910870
6	0	5.688591	-0.720291	-0.418742
1	0	5.253314	1.229202	0.381728
1	0	4.385303	-0.070722	1.165547
6	0	6.906850	-0.975364	0.437271
1	0	5.199000	-1.683978	-0.596088
1	0	6.044869	-0.388876	-1.400992
8	0	7.705872	-1.926477	-0.111098
8	0	7.176818	-0.426895	1.476158
1	0	8.465414	-2.038417	0.480299

TS8

6	0	-2.321665	-2.539866	-0.426321
6	0	-3.695279	-2.148965	-0.980285
6	0	-4.644821	-1.517188	0.061326
6	0	-3.995132	-0.330170	0.753463
8	0	-3.716054	0.664809	-0.132165
6	0	-2.942315	1.783141	0.325415
6	0	-1.676835	1.841087	-0.537526
8	0	-0.852650	0.732700	-0.380342
6	0	-0.663655	2.796897	-0.061506
6	0	-6.002652	-1.138326	-0.566295
6	0	-7.036214	-0.614301	0.435281
8	0	-3.757020	-0.257998	1.933395
8	0	1.203814	1.997828	-0.864094
6	0	2.280186	1.791138	0.054173
6	0	3.326945	0.847171	-0.535935
1	0	2.722812	2.771273	0.255619
1	0	-5.835264	-0.397104	-1.353902
1	0	-6.396217	-2.033757	-1.059869
1	0	-7.985773	-0.400140	-0.062741
1	0	-7.230435	-1.346028	1.225204
1	0	-6.701491	0.308492	0.917951
1	0	-4.818431	-2.241431	0.863687

1	0	-3.570214	-1.450869	-1.813897
1	0	-4.200745	-3.033942	-1.383251
1	0	-2.416439	-3.207684	0.435881
1	0	-1.737459	-3.062094	-1.189290
1	0	-1.749620	-1.659618	-0.120897
1	0	-3.553212	2.682259	0.200981
1	0	-2.688618	1.642572	1.376430
1	0	-1.963431	2.024433	-1.585272
1	0	-0.291807	3.634076	-0.635412
1	0	-0.392717	2.742225	0.985636
1	0	0.551921	1.218010	-0.820932
1	0	1.892244	1.387013	0.998338
6	0	4.510846	0.612977	0.410793
1	0	3.679694	1.265405	-1.484506
1	0	2.846062	-0.109012	-0.770631
6	0	5.566371	-0.327200	-0.185871
1	0	4.979001	1.574481	0.656796
1	0	4.145641	0.195467	1.357304
6	0	6.739368	-0.565217	0.764387
1	0	5.101143	-1.287063	-0.435590
1	0	5.939963	0.094026	-1.125871
16	0	7.969691	-1.685743	-0.039932

1	0	7.230273	0.379009	1.008256
1	0	6.392084	-1.029906	1.689191
1	0	8.840384	-1.733988	0.988749

TS9

6	0	-2.108827	-2.572585	-0.439137
6	0	-3.447829	-2.071367	-0.988804
6	0	-4.343092	-1.372513	0.058165
6	0	-3.600773	-0.246658	0.758951
8	0	-3.233651	0.722470	-0.121800
6	0	-2.385638	1.781114	0.347407
6	0	-1.127772	1.775067	-0.527102
8	0	-0.361848	0.623888	-0.384811
6	0	-0.060066	2.671331	-0.054072
6	0	-5.664692	-0.878847	-0.567236
6	0	-6.654818	-0.283293	0.438282
8	0	-3.365992	-0.197197	1.940882
8	0	1.758863	1.781816	-0.871728
6	0	2.827736	1.519712	0.042036
6	0	3.832532	0.537324	-0.557492
1	0	3.312208	2.478212	0.251342
1	0	-5.434971	-0.145429	-1.346279

1	0	-6.128266	-1.734283	-1.070956
1	0	-7.584773	0.007259	-0.058035
1	0	-6.905227	-1.003473	1.222944
1	0	-6.249914	0.607379	0.927505
1	0	-4.576058	-2.086491	0.854654
1	0	-3.269872	-1.381742	-1.819707
1	0	-4.022272	-2.911960	-1.394390
1	0	-2.254451	-3.229049	0.424679
1	0	-1.571333	-3.141426	-1.203052
1	0	-1.465863	-1.741312	-0.136898
1	0	-2.939008	2.719841	0.246550
1	0	-2.131234	1.605169	1.392904
1	0	-1.413682	1.981348	-1.570837
1	0	0.351411	3.490950	-0.626177
1	0	0.214711	2.596475	0.990777
1	0	1.070375	1.035666	-0.830219
1	0	2.423959	1.124482	0.983304
6	0	5.004629	0.243600	0.387421
1	0	4.203847	0.948566	-1.501956
1	0	3.311355	-0.395091	-0.801324
6	0	6.020891	-0.732084	-0.215798
1	0	5.509100	1.183894	0.644847

1	0	4.617816	-0.166180	1.329110
6	0	7.176113	-1.038984	0.725111
1	0	5.526892	-1.673665	-0.478197
1	0	6.429916	-0.321235	-1.145121
8	0	8.062369	-1.941474	0.059276
1	0	7.701116	-0.110538	0.991312
1	0	6.795069	-1.487727	1.653257
1	0	8.790838	-2.156580	0.649015

TS1-a

6	0	8.493472	1.414076	2.380193
6	0	8.327859	2.021158	0.984228
6	0	8.930795	1.169187	-0.153273
6	0	8.291857	-0.208560	-0.210711
8	0	6.958647	-0.128285	-0.453192
6	0	6.232173	-1.365796	-0.481150
6	0	4.757881	-1.001290	-0.437896
8	0	4.334772	-0.573985	0.834180
6	0	8.830682	1.891369	-1.514141
6	0	9.498182	1.148106	-2.674853
8	0	8.865631	-1.260489	-0.065821
6	0	3.812817	-2.119099	-0.393604

8	0	1.715948	-1.697613	0.389542
6	0	1.359958	-0.556128	0.756009
6	0	-0.101718	-0.273966	1.058139
6	0	-1.089713	-1.314888	0.531111
6	0	-2.525044	-0.957798	0.918338
6	0	-3.556968	-1.904170	0.357528
8	0	-3.372440	-2.756526	-0.476439
8	0	2.165311	0.429571	0.919560
8	0	-4.778350	-1.661784	0.906814
6	0	-5.889882	-2.400252	0.371366
6	0	-6.389585	-1.858740	-0.975837
8	0	-7.571457	-2.578666	-1.337587
6	0	-6.804150	-0.387690	-0.947221
8	0	-5.599002	0.392491	-0.934277
6	0	-5.423269	1.608928	-0.350258
8	0	-4.289479	2.016136	-0.283561
6	0	-6.598994	2.445126	0.173826
6	0	-7.130981	1.972448	1.549292
6	0	-6.050796	1.764481	2.615172
6	0	-7.731648	2.683563	-0.846039
6	0	-7.253572	3.236545	-2.192781
1	0	8.091739	2.086024	3.142910

1	0	9.547146	1.233121	2.613510
1	0	7.963547	0.462017	2.473008
1	0	7.267950	2.194034	0.776399
1	0	8.818582	3.000191	0.952855
1	0	9.298109	2.874989	-1.396608
1	0	7.775909	2.073567	-1.741917
1	0	9.027096	0.178899	-2.863465
1	0	9.431210	1.730999	-3.597315
1	0	10.557303	0.963507	-2.472110
1	0	9.986052	0.978092	0.066802
1	0	6.500172	-1.975031	0.383689
1	0	6.480444	-1.915820	-1.394785
1	0	3.991168	-2.904274	0.328370
1	0	3.039038	-2.279595	-1.126586
1	0	3.270351	0.010097	0.847602
1	0	-0.170549	-0.187842	2.150052
1	0	-0.324041	0.724356	0.669343
1	0	-1.013542	-1.384199	-0.556589
1	0	-0.828514	-2.303278	0.915555
1	0	-2.651321	-0.921120	2.005605
1	0	-2.796411	0.040096	0.551353
1	0	-6.681582	-2.311626	1.116458

1	0	-5.612037	-3.451131	0.261127
1	0	-5.610846	-1.977632	-1.734623
1	0	-7.323525	-3.455046	-1.648014
1	0	-7.369545	-0.172690	-1.856645
1	0	-7.430601	-0.184376	-0.079507
1	0	-6.116191	3.411357	0.348424
1	0	-8.422110	3.399932	-0.387248
1	0	-8.315034	1.772613	-1.002974
1	0	-7.833794	2.741132	1.889118
1	0	-7.724831	1.059318	1.443364
1	0	-6.507281	1.524613	3.579092
1	0	-5.376126	0.945439	2.354068
1	0	-5.444495	2.665001	2.744907
1	0	-8.101900	3.446566	-2.849134
1	0	-6.694279	4.167263	-2.060832
1	0	-6.598860	2.531727	-2.713447
1	0	4.528819	-0.277742	-1.230187

TS2-a

6	0	5.190655	2.805725	-0.507743
6	0	6.670918	2.978298	-0.861155

6	0	7.645194	2.640371	0.287022
6	0	9.110813	2.906050	-0.120156
6	0	10.127359	2.698424	1.006376
6	0	7.458683	1.214408	0.779363
8	0	7.153859	0.897351	1.901461
8	0	7.702779	0.317629	-0.216360
6	0	7.454350	-1.071085	0.041124
6	0	6.399907	-1.543030	-0.963535
6	0	5.889803	-2.899761	-0.735827
8	0	5.153687	-0.950516	-0.758850
16	0	3.469629	-2.863200	-2.074903
6	0	2.337846	-2.977565	-0.623980
6	0	1.096882	-2.092942	-0.760759
6	0	0.158259	-2.245489	0.443592
6	0	-1.084688	-1.363019	0.327142
6	0	-2.024099	-1.493408	1.506107
8	0	-1.815358	-2.124723	2.512895
8	0	-3.161927	-0.791918	1.295189
6	0	-4.122923	-0.798323	2.369987
6	0	-5.298527	0.083966	1.993979
6	0	-5.896417	-0.285106	0.643788
8	0	-7.042411	0.582593	0.462829

6	0	-7.757544	0.412186	-0.678048
8	0	-7.478732	-0.422642	-1.503299
8	0	-4.869113	1.439858	2.021554
6	0	-8.925063	1.380618	-0.777269
6	0	-10.249286	0.593871	-0.867555
6	0	-10.564596	-0.253565	0.368163
6	0	-8.734446	2.301715	-2.001291
6	0	-7.499095	3.203358	-1.927335
1	0	2.054054	-4.028172	-0.534365
1	0	9.362356	2.268915	-0.973781
1	0	9.168517	3.940602	-0.475796
1	0	11.138509	2.935611	0.664616
1	0	9.905568	3.339419	1.864839
1	0	10.133617	1.664303	1.362766
1	0	7.402510	3.265934	1.152056
1	0	6.919049	2.360793	-1.729764
1	0	6.861205	4.018021	-1.150760
1	0	4.919790	3.401361	0.369702
1	0	4.558355	3.130076	-1.338895
1	0	4.948183	1.760182	-0.298508
1	0	8.398690	-1.605584	-0.096048
1	0	7.095015	-1.194619	1.062831

1	0	6.786404	-1.401315	-1.983995
1	0	5.933713	-3.701726	-1.456831
1	0	5.534463	-3.134403	0.259349
1	0	4.160070	-1.664724	-1.624955
1	0	2.907798	-2.698987	0.264382
1	0	0.566655	-2.350085	-1.684171
1	0	1.410762	-1.048456	-0.860170
1	0	-0.150425	-3.291216	0.544458
1	0	0.690537	-1.996929	1.366607
1	0	-0.809971	-0.304910	0.246822
1	0	-1.651668	-1.588900	-0.582252
1	0	-4.454373	-1.824091	2.551046
1	0	-3.654653	-0.421307	3.280391
1	0	-6.065979	-0.079014	2.766033
1	0	-5.605358	1.987336	1.726573
1	0	-5.184543	-0.115818	-0.163787
1	0	-6.223636	-1.327268	0.619356
1	0	-8.933393	1.991107	0.131474
1	0	-9.633368	2.920445	-2.092163
1	0	-8.688061	1.678361	-2.899796
1	0	-11.054613	1.318312	-1.028567
1	0	-10.213863	-0.042670	-1.757093

1	0	-7.443004	3.855057	-2.802997
1	0	-7.524484	3.842726	-1.039339
1	0	-6.573127	2.621914	-1.895090
1	0	-11.530651	-0.753031	0.258393
1	0	-9.812486	-1.031796	0.527637
1	0	-10.608405	0.360514	1.272967

TS3-a

6	0	4.933358	-2.372513	1.317207
6	0	6.433076	-2.351000	1.628085
6	0	7.343322	-2.381534	0.381840
6	0	7.011660	-1.254389	-0.582829
8	0	7.198456	-0.038693	-0.000965
6	0	6.791488	1.131339	-0.725212
6	0	5.735114	1.848552	0.121897
8	0	4.567916	1.111373	0.285352
6	0	5.063796	2.960752	-0.570007
6	0	8.836421	-2.368625	0.773568
6	0	9.801408	-2.526613	-0.405034
8	0	6.644738	-1.399426	-1.722292
8	0	3.124458	3.103861	0.424251
6	0	1.969135	3.081361	-0.418070

6	0	0.712262	2.740603	0.381616
1	0	1.877037	4.076673	-0.862550
1	0	9.052985	-1.441680	1.313701
1	0	8.994821	-3.187276	1.484305
1	0	10.837670	-2.552021	-0.056997
1	0	9.610011	-3.453890	-0.953354
1	0	9.713064	-1.700992	-1.117174
1	0	7.136822	-3.296442	-0.182780
1	0	6.674056	-1.463269	2.221020
1	0	6.697385	-3.220006	2.241379
1	0	4.669356	-3.232589	0.693560
1	0	4.352896	-2.440951	2.241578
1	0	4.619775	-1.462043	0.799078
1	0	7.679252	1.754361	-0.870092
1	0	6.376830	0.834828	-1.689011
1	0	6.189002	2.162043	1.075412
1	0	5.066216	3.986206	-0.227976
1	0	4.679159	2.760872	-1.562447
1	0	3.461317	2.158318	0.585211
1	0	2.111544	2.353765	-1.227395
6	0	-0.547458	2.742467	-0.492374
1	0	0.610443	3.463844	1.197830

1	0	0.846756	1.756580	0.845287
6	0	-1.803620	2.396655	0.307158
1	0	-0.678944	3.722800	-0.960809
1	0	-0.434800	2.027788	-1.313927
6	0	-3.070833	2.424084	-0.520252
1	0	-1.722202	1.405919	0.766623
1	0	-1.944635	3.095031	1.139996
8	0	-4.131347	2.024908	0.215911
8	0	-3.153906	2.758783	-1.676868
6	0	-5.413917	2.044843	-0.442320
6	0	-6.482309	1.636616	0.554900
1	0	-7.419629	1.557049	-0.013978
6	0	-6.208797	0.297366	1.237560
8	0	-6.579914	2.667861	1.535436
1	0	-5.616118	3.050746	-0.813633
1	0	-5.399378	1.352730	-1.285962
1	0	-7.320328	2.478913	2.121042
1	0	-6.981466	0.079347	1.980028
8	0	-6.227221	-0.721679	0.217978
1	0	-5.241183	0.306825	1.739745
6	0	-6.096111	-2.004504	0.644060
6	0	-6.118405	-2.990077	-0.512834

8	0	-5.982195	-2.292860	1.810103
1	0	-6.001387	-3.971300	-0.041856
6	0	-7.470553	-2.955832	-1.257180
6	0	-4.935351	-2.760013	-1.477500
1	0	-5.018393	-3.502272	-2.278676
1	0	-5.045815	-1.779176	-1.950297
6	0	-3.556822	-2.873204	-0.820455
1	0	-7.410159	-3.677606	-2.078797
6	0	-8.681241	-3.285961	-0.379396
1	0	-7.598494	-1.970923	-1.717037
1	0	-9.601339	-3.279228	-0.969644
1	0	-8.583490	-4.275725	0.076153
1	0	-8.805010	-2.562857	0.432122
1	0	-2.763084	-2.741557	-1.560256
1	0	-3.413101	-2.115619	-0.044577
1	0	-3.419055	-3.852488	-0.352660

TS4-a

6	0	-7.446162	3.867079	-1.196147
6	0	-7.591205	3.410412	0.258251
6	0	-8.840423	2.543540	0.529096
6	0	-8.818372	1.274463	-0.305428

8	0	-7.768061	0.478380	0.023219
6	0	-7.625762	-0.732243	-0.733739
6	0	-6.329093	-1.378186	-0.284037
8	0	-5.183190	-0.734301	-0.800929
6	0	-8.996693	2.230897	2.032635
6	0	-10.260940	1.441207	2.383985
8	0	-9.614242	0.976877	-1.162483
6	0	-5.932144	-2.605049	-0.976407
8	0	-3.713080	-3.205933	-0.969159
6	0	-2.871184	-2.541798	-0.319981
6	0	-1.493452	-3.140987	-0.085998
6	0	-0.344533	-2.131630	-0.199877
6	0	1.017379	-2.770132	0.102165
6	0	2.167274	-1.769909	-0.021793
1	0	2.022889	-0.939446	0.674742
8	0	-3.104776	-1.390396	0.187559
16	0	3.767355	-2.588203	0.363021
1	0	-6.571313	4.511879	-1.312293
1	0	-8.324308	4.430243	-1.526118
1	0	-7.320817	3.020534	-1.877109
1	0	-6.699838	2.856566	0.567265
1	0	-7.656874	4.288805	0.909593

1	0	-9.006188	3.187952	2.565129
1	0	-8.109428	1.691417	2.378001
1	0	-10.267156	0.453518	1.913397
1	0	-10.336368	1.290768	3.464119
1	0	-11.162568	1.966818	2.055782
1	0	-9.728071	3.085639	0.187670
1	0	-7.587423	-0.503101	-1.800591
1	0	-8.480872	-1.388604	-0.543934
1	0	-6.309627	-1.468357	0.808038
1	0	-5.989470	-2.622539	-2.056011
1	0	-5.721154	-3.533000	-0.471297
1	0	-4.183397	-1.005526	-0.243678
1	0	-1.511361	-3.566823	0.925534
1	0	-1.364728	-3.974670	-0.779282
1	0	-0.335913	-1.707848	-1.210783
1	0	-0.531494	-1.299826	0.484081
1	0	1.009494	-3.186652	1.115728
1	0	1.192047	-3.610100	-0.579816
6	0	4.883587	-1.141330	0.169909
1	0	2.211944	-1.363427	-1.036698
6	0	6.335128	-1.589071	0.048181
1	0	4.595726	-0.605333	-0.738434

1	0	4.781862	-0.470439	1.023316
6	0	7.266129	-0.441298	-0.353085
8	0	6.710890	-2.172717	1.292484
1	0	6.407523	-2.336066	-0.755996
1	0	7.524956	-2.672814	1.178921
1	0	8.302118	-0.791332	-0.307904
1	0	7.042573	-0.136723	-1.381428
8	0	7.064710	0.657737	0.543662
6	0	7.806649	1.799449	0.521559
8	0	7.526143	2.665556	1.308320
6	0	8.960893	1.954875	-0.471972
1	0	9.023082	1.088077	-1.135091
6	0	8.721661	3.208331	-1.340515
6	0	10.288404	2.065484	0.311075
1	0	11.083469	2.279074	-0.411258
1	0	10.220433	2.931903	0.975175
6	0	10.654719	0.818682	1.121590
1	0	9.608324	3.352116	-1.967201
6	0	7.475222	3.132336	-2.226796
1	0	8.653998	4.076253	-0.678705
1	0	11.605131	0.962351	1.641806
1	0	9.899773	0.592255	1.880097

1	0	10.765128	-0.060244	0.477569
1	0	7.378032	4.034172	-2.836535
1	0	7.520651	2.277869	-2.910346
1	0	6.561901	3.043536	-1.631205

TS5-a

6	0	-4.004075	1.643635	2.074550
6	0	-5.440237	2.175865	2.096498
6	0	-5.904793	2.814275	0.770736
6	0	-5.752837	1.857105	-0.400352
8	0	-6.496265	0.731415	-0.214167
6	0	-6.371199	-0.331214	-1.169094
6	0	-5.816007	-1.555847	-0.432470
8	0	-4.522044	-1.363842	0.044207
6	0	-5.440585	-2.672742	-1.308102
6	0	-7.351090	3.345219	0.875350
6	0	-7.839274	4.098978	-0.365267
8	0	-5.084078	2.059612	-1.382755
16	0	-3.648009	-4.106303	0.182339
6	0	-2.110555	-3.669403	-0.737684
6	0	-0.888930	-3.547241	0.175377
1	0	-1.959910	-4.451593	-1.484809

1	0	-8.020616	2.506939	1.091858
1	0	-7.393294	4.010447	1.744740
1	0	-8.854138	4.477023	-0.214812
1	0	-7.194036	4.953386	-0.590444
1	0	-7.853759	3.457379	-1.250992
1	0	-5.241145	3.651118	0.530194
1	0	-6.129326	1.367888	2.361094
1	0	-5.539426	2.940344	2.875460
1	0	-3.295292	2.426772	1.786977
1	0	-3.715387	1.281810	3.065357
1	0	-3.896212	0.809953	1.374894
1	0	-7.369271	-0.529276	-1.570011
1	0	-5.696138	-0.024401	-1.968210
1	0	-6.529037	-1.854220	0.351743
1	0	-5.859762	-3.666046	-1.250686
1	0	-4.788196	-2.450296	-2.142592
1	0	-3.984547	-2.750525	0.505114
1	0	-2.301619	-2.726730	-1.253962
6	0	0.377006	-3.159284	-0.601950
1	0	-0.727370	-4.495900	0.698086
1	0	-1.089358	-2.795451	0.945587
6	0	1.614410	-3.050286	0.299213

1	0	0.564387	-3.899135	-1.390388
1	0	0.209779	-2.201205	-1.109322
6	0	2.858822	-2.604078	-0.469392
1	0	1.416299	-2.338570	1.108027
1	0	1.806318	-4.018554	0.774624
16	0	4.308216	-2.524022	0.659390
1	0	3.078329	-3.306481	-1.278721
1	0	2.699240	-1.614401	-0.907068
6	0	5.558837	-1.864298	-0.521603
6	0	6.707676	-1.137438	0.169709
1	0	5.968740	-2.687223	-1.110556
1	0	5.060814	-1.161875	-1.192533
1	0	7.406073	-0.824483	-0.620273
6	0	6.290770	0.104860	0.955667
8	0	7.342290	-2.068192	1.046326
1	0	8.114947	-1.654368	1.445052
1	0	7.156017	0.551877	1.453030
8	0	5.741696	1.053265	0.017130
1	0	5.543274	-0.140287	1.710952
6	0	5.463847	2.290406	0.500696
6	0	4.860673	3.193239	-0.564077
8	0	5.681759	2.607935	1.644320

1	0	4.695707	4.147898	-0.054461
6	0	5.837630	3.420316	-1.737009
6	0	3.499848	2.655636	-1.055759
1	0	3.126764	3.354012	-1.812720
1	0	3.658598	1.700218	-1.566044
6	0	2.450450	2.493488	0.047859
1	0	1.496917	2.160428	-0.370051
1	0	2.757694	1.758224	0.796783
1	0	2.273675	3.439205	0.568401
1	0	5.334097	4.068781	-2.462208
6	0	7.171623	4.053568	-1.330975
1	0	6.014753	2.465928	-2.242602
1	0	7.800237	4.229617	-2.207876
1	0	7.018760	5.014222	-0.830419
1	0	7.734200	3.412662	-0.645879

TS6-a

6	0	-6.300500	-1.255641	-2.527776
6	0	-7.815764	-1.148882	-2.331510
6	0	-8.374035	-2.034520	-1.196906
6	0	-7.695194	-1.732377	0.129345
8	0	-7.907215	-0.441692	0.504152

6	0	-7.211500	0.057495	1.655701
6	0	-6.360264	1.244198	1.193621
8	0	-5.333399	0.885005	0.328087
6	0	-5.451334	1.772686	2.223366
6	0	-9.908762	-1.910554	-1.086660
6	0	-10.546128	-2.844473	-0.053626
8	0	-7.060159	-2.521460	0.783608
8	0	-3.848823	2.705219	1.071131
6	0	-2.511088	2.326672	1.403698
6	0	-1.537692	2.704467	0.287858
1	0	-2.250792	2.853501	2.326826
1	0	-10.164843	-0.870977	-0.859952
1	0	-10.325623	-2.125554	-2.076701
1	0	-11.634662	-2.741301	-0.057025
1	0	-10.308658	-3.891453	-0.264723
1	0	-10.197588	-2.627160	0.960147
1	0	-8.120165	-3.078696	-1.406065
1	0	-8.091329	-0.107934	-2.137135
1	0	-8.329905	-1.443607	-3.253547
1	0	-5.993103	-2.293760	-2.689149
1	0	-5.986199	-0.675607	-3.399983
1	0	-5.757394	-0.866175	-1.662212

1	0	-7.964323	0.366095	2.387471
1	0	-6.582626	-0.730832	2.070225
1	0	-7.023123	2.022329	0.782932
1	0	-5.471376	2.787224	2.595913
1	0	-4.832065	1.054831	2.746878
1	0	-4.274719	2.013572	0.459623
1	0	-2.466039	1.247173	1.598675
6	0	-0.083797	2.366718	0.640698
1	0	-1.636767	3.776137	0.086243
1	0	-1.829645	2.180658	-0.629143
6	0	0.901036	2.743073	-0.474551
1	0	0.197351	2.889378	1.563779
1	0	0.002209	1.294007	0.854398
6	0	2.350877	2.425558	-0.105196
1	0	0.634176	2.209041	-1.392975
1	0	0.810329	3.811898	-0.696789
16	0	3.476258	2.909843	-1.476855
1	0	2.642804	2.975744	0.794715
1	0	2.461675	1.355893	0.094150
6	0	5.097482	2.519532	-0.719239
1	0	5.830975	3.116285	-1.265746
1	0	5.084160	2.870860	0.316091

6	0	5.514765	1.051692	-0.786670
6	0	6.686426	0.785695	0.151233
1	0	4.676949	0.411559	-0.477781
8	0	5.869662	0.768562	-2.134720
1	0	6.119380	-0.160227	-2.187198
8	0	7.027833	-0.612117	0.004587
1	0	7.553572	1.395083	-0.115217
1	0	6.422032	0.983312	1.192554
6	0	7.992805	-1.093580	0.830364
6	0	8.267441	-2.568398	0.583740
8	0	8.544827	-0.406339	1.653991
1	0	9.030581	-2.833105	1.322501
6	0	7.012667	-3.430722	0.836706
6	0	8.845409	-2.807329	-0.827757
1	0	7.284560	-4.472292	0.634293
6	0	6.448994	-3.320332	2.256263
1	0	6.242403	-3.162770	0.106857
1	0	9.017764	-3.884012	-0.931464
1	0	8.089040	-2.542597	-1.573666
6	0	10.147607	-2.053674	-1.112781
1	0	5.588882	-3.982733	2.383864
1	0	7.198010	-3.597358	3.003926

1	0	6.117682	-2.303069	2.484407
1	0	10.527777	-2.301417	-2.107152
1	0	10.007864	-0.969578	-1.074958
1	0	10.922133	-2.311108	-0.384408

TS7-a

6	0	-7.324947	3.660303	-1.252002
6	0	-7.612140	3.251126	0.195358
6	0	-8.860092	2.357452	0.365647
6	0	-8.726175	1.068595	-0.426964
8	0	-7.681413	0.318493	0.011445
6	0	-7.433795	-0.904260	-0.696403
6	0	-6.127096	-1.458882	-0.162313
8	0	-4.996995	-0.758548	-0.641919
6	0	-9.156397	2.076596	1.854533
6	0	-10.437472	1.274783	2.102192
8	0	-9.436978	0.720104	-1.337727
6	0	-5.618980	-2.674620	-0.799239
8	0	-3.365245	-3.132184	-0.692346
6	0	-2.593186	-2.395442	-0.034838
6	0	-1.171167	-2.865255	0.226476
6	0	-0.115747	-1.830018	-0.189360

6	0	1.310785	-2.296316	0.121274
6	0	2.355569	-1.276843	-0.306466
1	0	2.203463	-0.321141	0.215996
8	0	-2.925141	-1.258032	0.447961
8	0	3.650907	-1.786966	-0.005754
1	0	-6.457941	4.324020	-1.300332
1	0	-8.176152	4.187615	-1.693146
1	0	-7.108436	2.793881	-1.883170
1	0	-6.744620	2.734200	0.616079
1	0	-7.769098	4.148734	0.803241
1	0	-9.230190	3.045136	2.360518
1	0	-8.298598	1.559817	2.295781
1	0	-10.388641	0.279170	1.651200
1	0	-10.610578	1.142180	3.173398
1	0	-11.311326	1.781937	1.682673
1	0	-9.722997	2.866481	-0.075552
1	0	-7.355265	-0.707185	-1.767298
1	0	-8.258689	-1.603238	-0.525377
1	0	-6.157459	-1.518204	0.931366
1	0	-5.624730	-2.726367	-1.879240
1	0	-5.370118	-3.571524	-0.256663
1	0	-4.016603	-0.951374	-0.035801

1	0	-1.091898	-3.056911	1.303263
1	0	-1.020251	-3.813923	-0.291809
1	0	-0.212370	-1.632512	-1.263518
1	0	-0.326821	-0.886969	0.322188
1	0	1.418116	-2.481506	1.195937
1	0	1.519303	-3.244526	-0.386065
6	0	4.708579	-0.906970	-0.365628
1	0	2.279481	-1.079845	-1.386387
6	0	5.992275	-1.531933	0.169029
1	0	4.762996	-0.795256	-1.458879
1	0	4.557248	0.080710	0.083573
6	0	7.235499	-0.713923	-0.141166
8	0	5.919487	-1.718746	1.575746
1	0	6.128372	-2.506654	-0.325556
1	0	5.039831	-2.068713	1.764013
1	0	8.066828	-1.131072	0.430226
1	0	7.459483	-0.777241	-1.210758
8	0	6.996777	0.654947	0.226058
6	0	7.951754	1.623068	0.202790
8	0	7.623592	2.741365	0.505518
6	0	9.390406	1.284386	-0.195339
1	0	9.476910	0.232572	-0.479414

6	0	9.798939	2.146160	-1.409106
6	0	10.322309	1.541241	1.009892
1	0	11.353011	1.395332	0.669011
1	0	10.227608	2.592944	1.295408
6	0	10.052267	0.646014	2.222793
1	0	10.861210	1.963498	-1.603735
6	0	8.991520	1.867726	-2.680308
1	0	9.701618	3.199289	-1.131001
1	0	10.747855	0.876060	3.033971
1	0	9.039623	0.783630	2.612245
1	0	10.176328	-0.413304	1.975964
1	0	9.344112	2.488985	-3.507627
1	0	9.084036	0.822149	-2.992698
1	0	7.929173	2.087740	-2.538989

TS8-a

6	0	3.692226	-2.151278	1.459341
6	0	5.122348	-2.699336	1.480818
6	0	5.744116	-2.926509	0.087025
6	0	5.736917	-1.659207	-0.752369
8	0	6.432667	-0.660855	-0.140814
6	0	6.420693	0.641406	-0.741058

6	0	5.715812	1.596914	0.230553
8	0	4.376475	1.276101	0.429185
6	0	5.431068	2.923829	-0.329856
6	0	7.168607	-3.513291	0.197186
6	0	7.811641	-3.874540	-1.145059
8	0	5.212347	-1.542791	-1.831519
16	0	3.349449	3.872465	1.159126
6	0	2.020899	3.691906	-0.107021
6	0	0.677686	3.274148	0.494518
1	0	1.937885	4.657168	-0.611045
1	0	7.802631	-2.802516	0.736597
1	0	7.104388	-4.410395	0.822553
1	0	8.798650	-4.320125	-0.994110
1	0	7.200421	-4.594411	-1.697515
1	0	7.939991	-2.996031	-1.783720
1	0	5.117566	-3.630048	-0.470685
1	0	5.768060	-2.023047	2.049411
1	0	5.140977	-3.662586	2.003314
1	0	3.026544	-2.803248	0.884555
1	0	3.293842	-2.082118	2.475433
1	0	3.654367	-1.150329	1.020364
1	0	7.461802	0.935157	-0.901279

1	0	5.887552	0.598347	-1.690929
1	0	6.295318	1.647344	1.165644
1	0	5.797479	3.853877	0.078491
1	0	4.925094	2.960583	-1.285951
1	0	3.695723	2.486363	1.165760
1	0	2.365227	2.949549	-0.829582
6	0	-0.403421	3.096142	-0.581009
1	0	0.354687	4.022607	1.225662
1	0	0.805697	2.335094	1.042399
6	0	-1.760348	2.689498	0.005061
1	0	-0.513659	4.030726	-1.145635
1	0	-0.071322	2.337628	-1.300869
6	0	-2.814721	2.456265	-1.066814
1	0	-1.653360	1.773368	0.595489
1	0	-2.123127	3.464954	0.687842
8	0	-4.041001	2.120539	-0.436483
1	0	-2.947343	3.360199	-1.681672
1	0	-2.503185	1.642156	-1.740526
6	0	-5.079432	1.825836	-1.349537
6	0	-6.384458	1.592609	-0.602741
1	0	-5.228705	2.658784	-2.050948
1	0	-4.834244	0.924126	-1.928683

1	0	-7.102304	1.190210	-1.331754
6	0	-6.249494	0.606137	0.555283
8	0	-6.836939	2.853706	-0.108841
1	0	-7.692271	2.736221	0.317141
1	0	-7.191700	0.522247	1.104711
8	0	-5.913599	-0.680934	-0.005004
1	0	-5.469153	0.926726	1.244677
6	0	-5.797951	-1.708454	0.871261
6	0	-5.410951	-3.004675	0.175892
8	0	-5.990429	-1.581873	2.056192
1	0	-5.326425	-3.737761	0.984537
6	0	-6.511813	-3.470339	-0.800939
6	0	-4.043659	-2.885652	-0.529419
1	0	-3.833021	-3.851764	-1.001134
1	0	-4.124880	-2.154311	-1.339535
6	0	-2.886249	-2.509485	0.399952
1	0	-6.163889	-4.397791	-1.268690
6	0	-7.875097	-3.712454	-0.146583
1	0	-6.608664	-2.733566	-1.604680
1	0	-1.940629	-2.487794	-0.148024
1	0	-3.030445	-1.522073	0.847466
1	0	-2.781992	-3.229921	1.216742

1	0	-8.598541	-4.075502	-0.881478
1	0	-7.806094	-4.457599	0.651373
1	0	-8.282822	-2.797916	0.293628

TS9-a

6	0	-4.947442	-2.216328	-1.521480
6	0	-6.469700	-2.254255	-1.685679
6	0	-7.252855	-2.378171	-0.361767
6	0	-6.894773	-1.268490	0.613591
8	0	-7.169188	-0.044048	0.086578
6	0	-6.766994	1.119102	0.824149
6	0	-5.768449	1.892651	-0.043753
8	0	-4.585902	1.198121	-0.271730
6	0	-5.106794	3.007303	0.654015
6	0	-8.775764	-2.434546	-0.610940
6	0	-9.614166	-2.688069	0.645414
8	0	-6.445306	-1.433004	1.720290
8	0	-3.197595	3.229734	-0.376175
6	0	-2.025552	3.199905	0.442660
6	0	-0.781402	2.881888	-0.385409
1	0	-1.933193	4.187639	0.904060
1	0	-9.091080	-1.502491	-1.090312

1	0	-8.958301	-3.234883	-1.336517
1	0	-10.676315	-2.756806	0.394948
1	0	-9.324495	-3.623271	1.133759
1	0	-9.499707	-1.886001	1.380287
1	0	-6.947051	-3.299368	0.144674
1	0	-6.806063	-1.356507	-2.213460
1	0	-6.753562	-3.109719	-2.309318
1	0	-4.585778	-3.091538	-0.972141
1	0	-4.457712	-2.211807	-2.499327
1	0	-4.624896	-1.318051	-0.987760
1	0	-7.667127	1.709083	1.021311
1	0	-6.303331	0.810927	1.761659
1	0	-6.269204	2.215780	-0.970125
1	0	-5.150586	4.040049	0.337488
1	0	-4.687970	2.796747	1.630255
1	0	-3.517068	2.282741	-0.564375
1	0	-2.148250	2.455992	1.240729
6	0	0.496448	2.864694	0.462743
1	0	-0.692690	3.623086	-1.186449
1	0	-0.918619	1.907827	-0.868035
6	0	1.748878	2.538543	-0.358995
1	0	0.622650	3.839999	0.950088

1	0	0.387094	2.129167	1.269571
6	0	3.014882	2.520017	0.484722
1	0	1.635253	1.561905	-0.841180
1	0	1.873049	3.274040	-1.160912
8	0	4.119640	2.208175	-0.352087
1	0	3.174487	3.499312	0.962387
1	0	2.932603	1.771615	1.287858
6	0	5.356300	2.171541	0.338407
6	0	6.448313	1.815934	-0.665571
1	0	5.589427	3.150365	0.778430
1	0	5.335904	1.426885	1.144054
8	0	7.678452	1.931812	0.047455
6	0	6.261400	0.450452	-1.311125
1	0	6.406874	2.550865	-1.482556
1	0	8.405631	1.700284	-0.538812
1	0	7.077728	0.230596	-2.004988
8	0	6.225642	-0.560873	-0.286028
1	0	5.324309	0.431890	-1.871937
6	0	6.339047	-1.846907	-0.692046
6	0	6.286300	-2.813367	0.480982
8	0	6.468003	-2.161941	-1.851294
1	0	6.410707	-3.803777	0.031386

6	0	7.444313	-2.566288	1.470956
6	0	4.915923	-2.761003	1.190054
1	0	7.334848	-3.285857	2.289758
6	0	8.838660	-2.708087	0.853959
1	0	7.330692	-1.570625	1.910039
1	0	4.952131	-3.468194	2.025919
1	0	4.781593	-1.767214	1.628560
6	0	3.726124	-3.101294	0.287927
1	0	9.614006	-2.561547	1.610538
1	0	8.981120	-3.700400	0.415556
1	0	9.008230	-1.971545	0.063181
1	0	2.791943	-3.084659	0.855545
1	0	3.623793	-2.389726	-0.536369
1	0	3.832769	-4.097965	-0.150408
